# Association of Reduced Maternal Plasma Cholinesterase Activity With Preeclampsia: A Meta-Analysis

**DOI:** 10.7759/cureus.47220

**Published:** 2023-10-17

**Authors:** Fouad K Mohammad, Ammar A Mohammed, Hishyar M Garmavy, Hussein M Rashid

**Affiliations:** 1 Department of Physiology, Biochemistry and Pharmacology, College of Veterinary Medicine, University of Mosul, Mosul, IRQ; 2 Department of Pharmacology, College of Pharmacy, University of Duhok, Duhok, IRQ

**Keywords:** butyrylcholinesterase, biomarker, preeclampsia, cholinesterase, pregnancy hypertension

## Abstract

Blood butyrylcholinesterase (BChE) activity has been found to decrease during pregnancy and reportedly decrease even more in preeclampsia (PE). The purpose of the present meta-analysis was to answer a specific question of whether BChE activity (in the plasma, serum, or whole blood) is reduced in pregnant women suffering from PE compared to those with normal pregnancy. The meta-analysis included 15 studies with 20 records of BChE activity in 608 women compared to 569 healthy pregnant (control) ones. The studies were subjected to quality assessment using the Newcastle-Ottawa Scale (NOS). Using the Meta-Essentials software program 1.5, the one-group random effects model and forest plot revealed that the percentage of BChE activity in pregnant women with PE was 84.84% of the control value, with a standard error of 4.09 and 95% C.I. of 76.28, 93.41, indicating a significant 15.16% reduction in BChE activity in comparison to healthy pregnancy. No significant heterogeneity was seen in the analyzed data and the funnel plot did show publication bias. Subgroup (mild, severe, and unclassified PE) forest plot analysis revealed that the % BChE activities in PE compared to respective healthy pregnancies were 96.28%, 97.08%, and 76.62%, respectively with no heterogeneity. The median NOS score of the 15 studies included in the meta-analysis was 7, ranging from 5 to 8 (medium to high quality), and the forest plot showed an effect size of 0.735. This meta-analysis shows that BChE activity is reduced in PE compared with normal pregnancy and its value as a biomarker warrants further clinical studies.

## Introduction and background

One of the most serious health consequences of pregnancy-induced hypertension is preeclampsia (PE) which affects about 5% to 8% of women in advanced pregnancy [1]. It is characterized by elevated maternal blood pressure (systolic > 140, diastolic > 90 mmHg) with > 0.3 g proteinuria in 24 h urine collection; in severe cases of PE the systolic and diastolic blood pressures may exceed 160 and 110 mmHg, respectively, with 24 h urine proteinuria of > 5 g, as well as disorders involving one or more of the major organ systems of the body [1,2]. In addition to these clinical findings, accumulated evidence from many studies has suggested that several biochemical changes could be considered biomarkers of PE [3-5]. These include but are not limited to elevation of activities of liver function enzymes [5], increased serum uric acid [6], hepcidin [7] and cytokine [8] levels, Vit D3 deficiency [9] or changes in blood cholinesterase (ChE) activity [10,11] and increased oxidative stress condition in the blood [12].

It is well known that pregnancy, especially in the period close to delivery, induces various physiologic and hemodynamic alterations which might impact responses to various drugs, muscle relaxants, or general anesthetics [13]. Pseudocholinesterase (E.C.3.1.1.8), also called butyrylcholinesterase (BChE), is found in the plasma, serum, or whole blood, and it was reported among other blood biochemical determinants such as oxidative stress, to undergo considerable alterations in the last trimester of pregnancy [11,14-16]. Hence, an additional reduction in BChE activity could be a risk factor associated with PE, especially when applying neuromuscular blocking agents in case anesthesia is needed for cesarean section delivery, stressing the fact that several studies have cautioned from the consequences of reduced BChE activity in pregnant women with PE [16-22]. However, discrepancies have emerged regarding the status of BChE activity in preeclamptic pregnant women. In some studies, in comparison to healthy pregnancy, investigators either did not find significant changes in BChE activity in PE [23], or they reported an increase [11] or even a decrease [24] in this enzyme activity. This enzyme is primarily synthesized in the liver, and it is released into the plasma [25,26]. It hydrolyzes butyrylcholine, acetylcholine, and other choline esters [22,25], and it has been known to modulate growth and ghrelin-driven activities such as weight gain [26]. BChE is also involved in maintaining neuronal integrity and participates in cellular adhesions, and it was reported to be reduced as a marker of systemic inflammation during cardiovascular ailments and endocrine dysfunctions [27]. This enzyme is a highly sensitive indicator of exposure to organophosphate and carbamate pesticides [25].

To further elaborate on the possibility of the occurrence of reduced BChE activity biomarkers in pregnant women suffering from PE, the purpose of the present meta-analysis was to accumulate evidence and analyze the data from published literature to answer this specific question of whether there would be reduced BChE activity (in the plasma, serum, or whole blood) in pregnant women suffering from PE compared to those with normal pregnancy.

## Review

Review method

Ethical Approval

The Reviewing Board, College of Pharmacy, University of Duhok, Iraq has approved to conduct of this meta-analysis on scientific publications reporting BChE activity in women with PE. None of the studies included in the meta-analysis were conducted in Iraq.

Registration

This systematic review and meta-analysis was registered on July 13, 2023 (reference No. CRD42023441616) at PROSPERO which is an international database of prospectively registered systematic reviews (National Institute of Health and Care Research, University of York, UK).

Study Period and Location

We conducted this meta-analysis from July 1, 2023 to August 31, 2023 at the College of Pharmacy, University of Duhok, Iraq.

Search Strategy

A literature search was done till August 31, 2023 using PubMed, Google Scholars, ScienceDirect, Directory of Open Access Journals, and Semantic Scholars for scientific publications reporting in English BChE activity in women with PE. The related searching keywords included ChE, plasma ChE, serum ChE or BChE, and PE, pregnancy-induced PE, pregnancy-induced hypertension, and toxemia. We also manually screened references of articles reporting BChE and PE.

Selection Criteria

**Figure 1:**
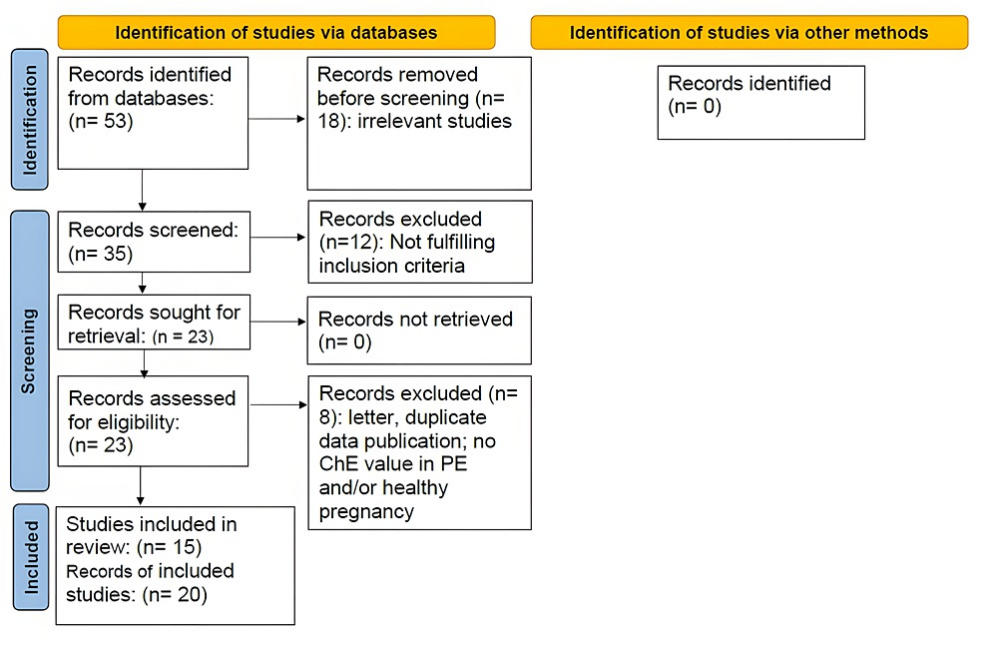
The Preferred Reporting Items for Systematic Reviews and Meta-Analysis flow diagram of databases searching, screening and including studies that reported butyrylcholinesterase activity in women with preeclampsia in comparison with healthy pregnancy.

The studies were selected in accordance with the Preferred Reporting Items for Systematic Reviews and Meta-Analysis (PRISMA) [28] as shown in Figure 1.

Inclusion Criteria

The framework of inclusion criteria included studies in pregnant women reporting the occurrence of PE (or toxemia) according to the American College of Obstetricians and Gynecologists (ACOG), with the mean ± standard deviation (SD) or the standard error (SE) or the range (minimum to maximum) of BChE activity reported in the serum, plasma or the whole blood in comparison with healthy pregnant (control) subjects, regardless of the number of participants or statistical significance of the changes in enzyme activity. When PE was classified into mild or severe in any study, both of their BChE data were included in the meta-analysis rather than the total one. In one instance, a study considered the ChE of healthy women as a control group and it was not included in the analysis [10]. In another instance, we calculated the SD of serum ChE activity from the range value reported [29] according to Wan et al. [30].

Exclusion Criteria

Exclusion criteria were concerned with studies in women with pregnancy hypertension not reporting the occurrence of PE, studies without data on BChE activity in preeclamptic women, or studies not having a proper control group (healthy pregnancy).

Data Extraction and Handling

All the reviewers (FKM, AAM, HMSG, and HMR) independently searched the databases mentioned above and obtained full-text articles for further evaluation according to inclusion and exclusion criteria. Initially, the reviewer FKM screened the studies for BChE activity in women with PE and tabulated the related information that included author name(s), year of publication, PE criteria as defined in the study itself, BChE activity, method of ChE determination and the unit of measurement, as well the number of participants in the groups. The BChE activity was found to be reported as mean, median, or range with the SD or SE. Values of BChE activity (mean ± SD) were extracted from the texts, tables, and figures of the studies. Thereafter, the rest of the reviewers (AAM, HMSG, and HMR) reviewed the studies according to inclusion and exclusion criteria and examined all the values of BChE activity. Any discrepancy about the inclusion or exclusion of studies or data extraction was resolved by consensus and agreement was achieved with the reviewer FKM. Figure 1 depicts all the studies screened and then selected for the present meta-analysis according to PRISMA guidelines [28]. Fifteen studies were selected [11,17,23,24,29,31-40], comprising 20 records of BChE activity in preeclamptic women in comparison with healthy pregnant controls.

Data Synthesis and Statistical Analysis

As shown in Table 1, various methods were used to determine BChE activity in studies that were selected for the present meta-analysis. Furthermore, the units of measurement of the enzyme activity varied considerably among the studies. To overcome this dilemma and in order to unify changes in the BChE activity across all the studies we converted the BChE activity in women with PE into the percentage of its corresponding activity in healthy pregnant (control) women (Table 2). The SD of BChE activity was also converted into percent of its respective mean (Table 2). Thus, we obtained a single group data showing the percentages (± SD) of BChE activities in women with PE relative to those of their corresponding control counterparts.

**Table 1:** Preeclampsia criteria in studies reporting butyrylcholinesterase activity in pregnant women ACOG: The American College of Obstetricians and Gynecologists

Authors [reference No.]	Age of preeclamptic women (years)	Blood sample	Criteria of preeclampsia (PE)	Method of cholinesterase determination
Parviainen et al., 1950 [29]	Not available	Serum	Pregnancy toxemia	Direct titration method of Davies et al., 1948
Pritchard, 1955 [31]	Not available	Plasma	Not available	Michel electrometric method
Robertson, 1966 [32]	Not available	Serum	PE (toxemia) hypertension and albuminuria, with or without edema	Radiometer pH-stat and titragraph method
Satyanarayana, 1986 [33]	22-26	Serum	Not available	Colorimetric method of De La Huerga et al., 1952
Kambam et al., 1987 [34]	18-40	Plasma	Not available	Ellman et al., 1961 spectrophotometric method
Kambam et al., 1988 [17]	18-38	Plasma	Not available	Ellman et al., 1961 spectrophotometric method
Garmendia et al., 1997 [35]	22 ± 7	Serum	ACOG classification of hypertension, 1986. Mild PE: systolic BP >= 140 mmHg, diastolic BP >=90 mmHg, with mild proteinuria or edema.	Rappaport method (kit, Sigma Chemical Co.)
Garmendia et al., 1997 [35]	23 ± 6	Serum	ACOG classification of hypertension, 1986. Severe PE: one or more of the following: systolic BP >= 160 mmHg, diastolic BP >=100 mmHg; proteinuria>2mg/24 h, increased serum creatinine level >177 µmol/L or oligouria <500 ml/24 h	Rappaport method (kit, Sigma Chemical Co.)
Mahmoud et al., 2003 [36]	28.13 ± 1.5	Serum	Sustained elevation of BP >= 140 ⁄ 90 mmHg, with proteinuria of 300 mg or more per 24 h	Ellman et al., 1961 spectrophotometric method
Harma et al., 2004 [23]	31.38 ± 6.9	Plasma	ACOG guidelines	Colorimetric method (kit, Rocholinesterase, Germany)
Osinubi et al., 2009 [24]	18-39	Plasma	Mild PE: BP 140 to <160 mmHg (or diastole hypertension of 90 to <110 mmHg) with proteinuria of 2+ (100 mg/dl)	Colorimetric method (kit, Randox Laboratories Ltd., UK)
Osinubi et al., 2009 [24]	18-39	Plasma	Severe PE: two of the following: systolic blood pressure > or =160 mmHg or diastolic blood pressure > or =110 mmHg; proteinuria (>3+ on dipstick [500 mg/dl]); facial edema	Colorimetric method (kit, Randox Laboratories Ltd., UK)
Kurdoglu et al., 2012 [37]	30.32 ± 7.02	Whole blood	Mild PE: systolic blood pressure of >= 140 mm Hg or diastolic blood pressure of >=90 mm Hg and 24 h proteinuria excretion >= 300 mg protein or >=1+	Ellman et al., 1961 spectrophotometric method
Kurdoglu et al., 2012 [37]	29.13 ± 4.91	Whole blood	Severe PE: one or more of systolic blood pressure of > = 160 mm Hg or diastolic blood pressure of >=110 mm Hg; proteinuria of >=2 g in a 24-hour urine specimen or >2+ on two random urine samples, serum creatinine of >= 1.2 mg/dl, elevated platelets, lactate dehydrogenase, serum transaminase levels, persistent headache	Ellman et al., 1961 spectrophotometric method
Rahimi et al., 2013 [38]	29.0 ± 5.7	Serum	Mild PE: systolic blood pressure >=140 mmHg, diastolic blood pressure >= 90 mmHg, 24 h proteinuria >300 mg, a urine protein: creatinine ratio of >0.3, >= 30 mg/dl protein in urine (1+‏ reaction on a standard urine dipstick)	Whittaker method
Rahimi et al., 2013 [38]	29.3 ± 6.4	Serum	Severe PE BP>= 160/110mmHg, proteinuria > 3+ or, headache, visual disturbances, upper abdominal pain, serum creatinine and transaminase elevation, thrombocytopenia, fetal-growth restriction	Whittaker method
Kharb et al., 2016 [39]	25.48 ± 3.96	Serum	Systolic blood pressure reading>=140 mmHg or diastolic blood pressure>= 90 mmHg, with or without proteinuria	Kinetic method (new DGKC method) using auto-analyzer
Inangil et al., 2016 [40]	27.3 ± 3.6	Plasma	BP 140/90 – 160/110; diastolic BP increased 15 mmHg and systolic BP increased > or = 30 mmHg from baseline of pre-pregnancy; or proteinuria > 300 mg/24 urine	Ellman et al. spectrophotometric method
Chen et al., 2022 [11]	30 ± 4	Serum	According to ACOG, mild PE: systolic blood pressure >=140mmHg and/or diastolic blood pressure >= 90mmHg, presence of 24 h proteinuria >=300mg, or urine dipsticks of 1‏+ protein	Not available
Chen et al., 2022 [11]	30 ± 4	Serum	According to ACOG severe PE: systolic blood pressure reading> or =160 mmHg or diastolic blood pressure> or = 110 mmHg, with or without proteinuria > or = 5 g in 24 h urine, or involvement of organs or systems	Not available

**Table 2:** The reported butyrylcholinesterase activity in pregnant women with preeclampsia Data of butyrylcholinesterase activity (mean ± SD) were extracted or calculated from the texts, tables or figures of studies reporting the enzyme activity of pregnant women with preeclampsia in comparison to healthy pregnant controls. Records A to T were included in the meta-analysis. Butyrylcholinesterase represents cholinesterase activity measured in the plasma, serum or whole blood. Records of mild (M) or severe (S) forms of preeclampsia were included in the meta-analysis whenever they were available in the study instead of the total record.

Authors [reference No.]	Healthy non-pregnant	n	Healthy pregnant	n	Preeclampsia	n	% of normal pregnancy
A-Parviainen et al., 1950 [29]	2.93 (2.58-3.36) cc. 0.01 N NaOH	6	2.37 (0.92-3.39)	13	2.6 ± 0.9 (1.48-5.08)	21	109.7 ± 34.6
B-Pritchard, 1955 [31]	0.84 ± 0.104 Δ pH	34	0.66 ± 0.103	49	0.68 ± 0.155	17	103 ± 22.8
C-Robertson 1966 [32]	208. 98 ± 38.9 unit	40	172.50 ± 39.7	111	166.30 ± 56.3	33	96.4 ± 33.9
D- Satyanarayana, 1986 [33]	190 ± 34.9 U/ml	15	254 ± 26.8	8	140 ± 22.1	10	55.1 ± 15.8
E-Kambam et al., 1987 (34)	438 ± 8l u/ml	11	257 ± 25	11	173 ± 18	11	67.3 ± 10.4
F-Kambam et al., 1988 [17]	426 ± 85 u/ml	15	264 ± 24	15	179 ± 25	15	67.8 ± 14.0
G-Garmendia et al., 1997 [35]			80 ± 22 U/ml	20	80 ± 16 M	16	100 ± 20
H- Garmendia et al., 1997 [35]					88 ± 16 S	17	110 ± 18.2
I-Mahmoud et al., 2003 [36]	0.98 ± 0.40 U	8	1.26 ± 0.35	15	1.17 ± 0.0.467	18	92.9 ± 39.9
J- Harma et al., 2004 [23]	8800.6 ± 3093.12 u/ml	30	7379.04 ± 2380.63	44	7063.03 ± 2115.55	32	95.7 ± 30.0
K- Osinubi et al., 2009 [24]	3594 ± 1042 m/L	30	2135 ± 422	30	1781 ± 330 M	30	83.4 ± 18.5
L- Osinubi et al., 2009 [24]					1630 ± 326 S	27	76.3 ± 20
M-Kurdoglu et al., 2012 (37)			12.58 ± 3.09 U/g Hb	50	11.65 ± 3.21 M	31	92.6 ± 27.6
N- Kurdoglu et al., 2012 [37]					10.11 ± 2.98 S	30	80.4 ± 29.5
O- Rahimi et al., 2013 [38]			690 ± 206 U/L	101	605 ± 201 M	128	87.7 ± 33.2
P- Rahimi et al., 2013 [38]					635 ± 217 S	70	92.0 ± 34.2
					616 ± 207 Total	198	89.3 ± 33.6
Q- Kharb et al., 2016 [39]	8167.028 ± 533.988 U/L	25	7972.2 ± 1544.15	25	7011.8 ± 1073.12	25	88.0 ± 15.3
R- Inangil et al., 2016 [40]			5580 ± 872 U/L	10	5326 ± 1129	10	95.4 ± 21.2
S- Chen et al., 2022 [11]			6212.84 ± 925.94 U/L	67	7223.38 ± 1593.09 M	23	116.3 ± 22.1
T- Chen et al., 2022 [11]					6639.05 ± 1089.95 S	44	106.9 ± 16.4
Chen et al., 2022 [11]					6840.21 ± 1302.51 Total	67	110.1 ± 19.0

The data of percent BChE activity in PE were subjected to a single group (one-arm) meta-analysis using the random effects model using the software program Meta-Essentials, Version 1.5 (https://www.erim.eur.nl/research-support/meta-essentials/download/). The meta-analysis included building forest and funnel plots and calculating effect size, weighted means with their 95% confidence intervals (C.I.) as well as a percentage weight of each study and the Z-test values at p < 0.05 [41]. The analysis output also included heterogeneity test results, publication bias, and subgroup analysis.

Heterogeneity Analysis

The possibility of the existence of heterogeneity in the analyzed data was assessed by the Cochrane Q-test at a p-value of < 0.10 [41]. Furthermore, another assessment of the existence of heterogeneity was conducted by calculating the I^2^; which results in values ranging from 0% (no heterogeneity) to 100% (high heterogeneity) [41].

Publication Bias

The funnel plot is a scatter plot in which the y-axis (inverse standard error) represents the precision and the x-axis shows the size effect [42]. We examined the forest plot visually for any publication bias; additionally, Egger’s statistical test was used to objectively test for bias.

Subgroup Analysis

Records of the 15 studies reporting BChE activity in mild, severe, or unclassified cases of PE (total n=20) were subjected to subgroup analysis [41,42].

Quality of the Studies

The studies of the present meta-analysis were subjected to quality assessment using the Newcastle-Ottawa Scale (NOS) to detect the risk of bias that might have occurred during the study [43]. At first two reviewers independently assessed the studies, and the final assessment was achieved collectively by the reviewers. The scoring system for each study included three quality variables, selection, comparability, and exposure, with a maximum score of 9 [43]. We considered any study with a score of < 5 of low quality, to be at a high risk of bias [43]. Furthermore, the scores of the 15 studies were subjected to one-group proportions meta-analysis using the online software: https://www.rbiostatistics.com/one_group_proportions.

Results

The studies selected for the present meta-analysis were according to the PRISMA flow chart (Figure 1). The final number of studies included in the meta-analysis was 15, comprising 20 records of BChE activity in pregnant women with PE (608), compared to healthy pregnant women (569) (Table 2). These studies were published between 1950 and 2022 (Table 1). The age of the preeclamptic women ranged between 18 and 40 years (Table 1).

Meta-analysis

**Figure 2:**
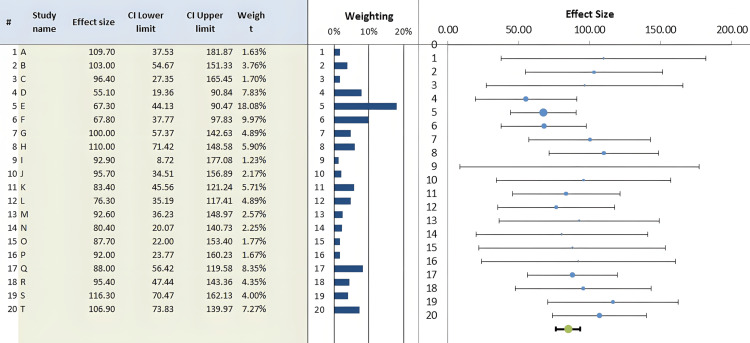
Forest plot of %butyrylcholinesterase activity in pregnant women with preeclampsia compared to healthy pregnant controls (15 studies, 20 records).

**Figure 3:**
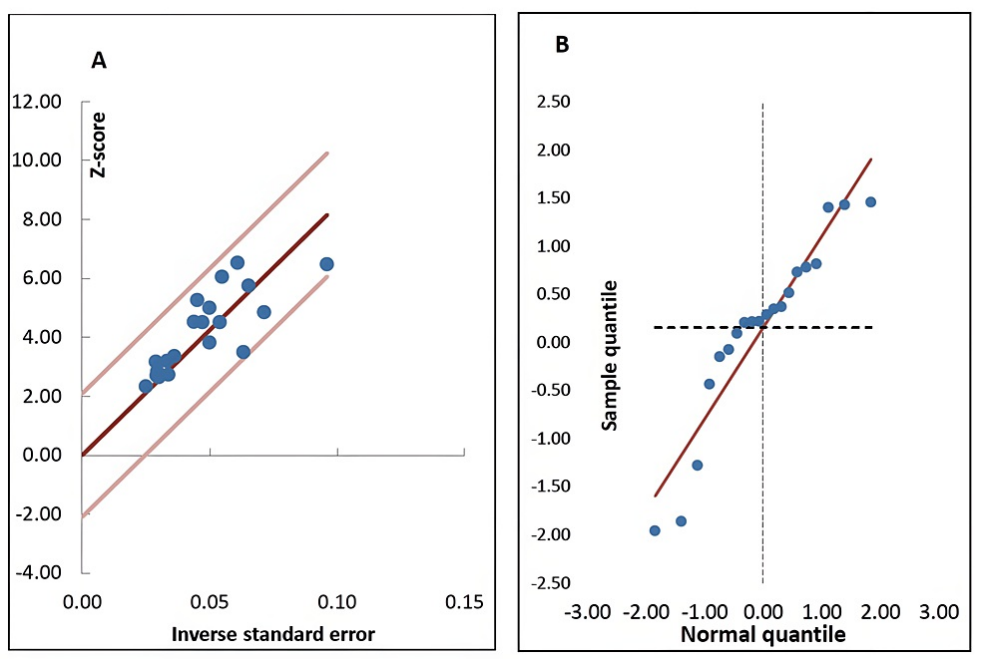
Galbraith (A) and quantile (B) plots of %butyrylcholinesterase activity in pregnant women with preeclampsia compared to healthy pregnant controls in 20 records from 15 studies.

Using the Meta-Essentials software program 1.5, the one-group random effects model and forest plot revealed that the percentage of BChE activity in pregnant women with PE was 84.84% of the control value, with a standard error of 4.09 and 95% C.I. of 76.28, 93.41 (Figure 2), indicating a significant (z= 20.74, p < 0.0001) 15.16% reduction in BChE activity. The percentages of weight of individual studies as shown by the analysis ranged between 1.23% and 18.08%, with the second largest value being 9.97% (Figure 2). As a supplement to the forest plot, the Galbraith plot showed that the data points of BChE activity were within the 95% C.I. lines parallel to the regression line, indicating neither heterogeneity nor potential outliers, and in the quantile plot, the data points relatively fell about a straight line (Figures 3A, 3B). This was also confirmed by the heterogeneity statistical analysis (Cochrane Q-test) of the data which did not show a significant effect as the Q value was 16.26 (p= 0.64) and the I^2^ index value was 0%, with respective zero values for T^2^ and T.

**Figure 4:**
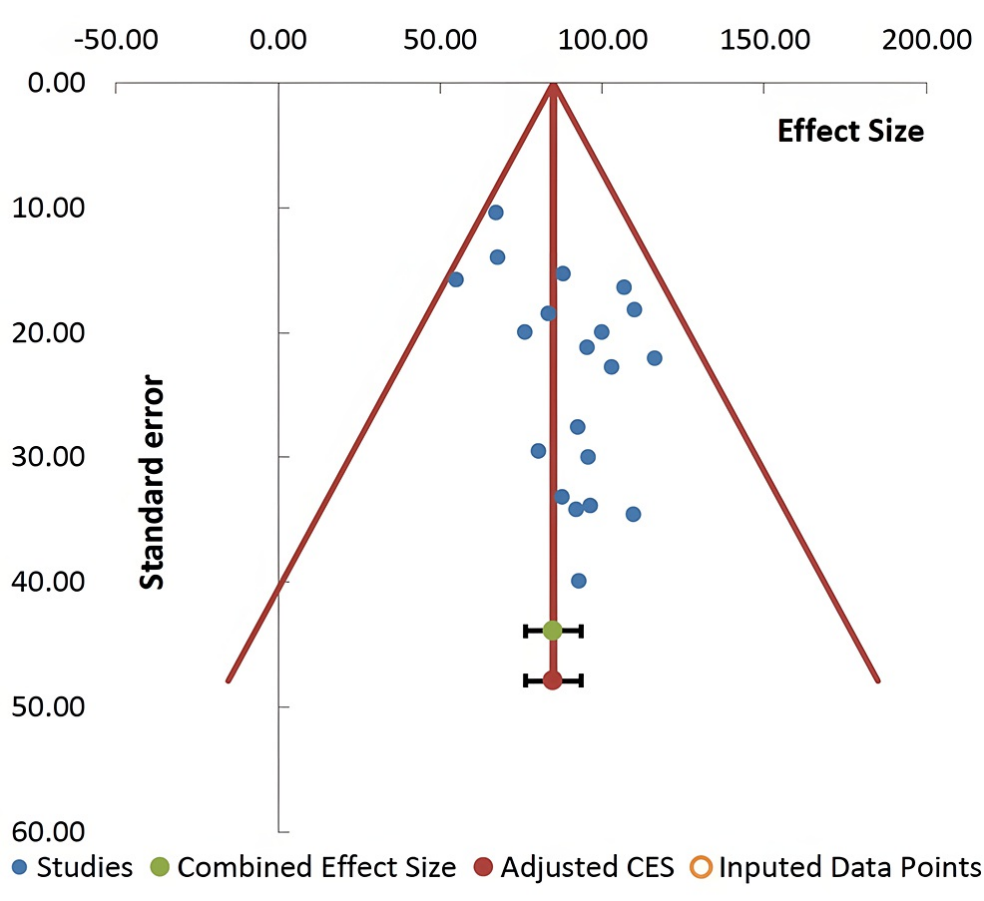
The funnel plot (publication bias) of %butyrylcholinesterase activity in pregnant women with preeclampsia compared to healthy pregnant controls in 20 records from 15 studies. The trim-and-fill analysis which included the number of the imputed points also indicated no missing studies.

**Figure 5:**
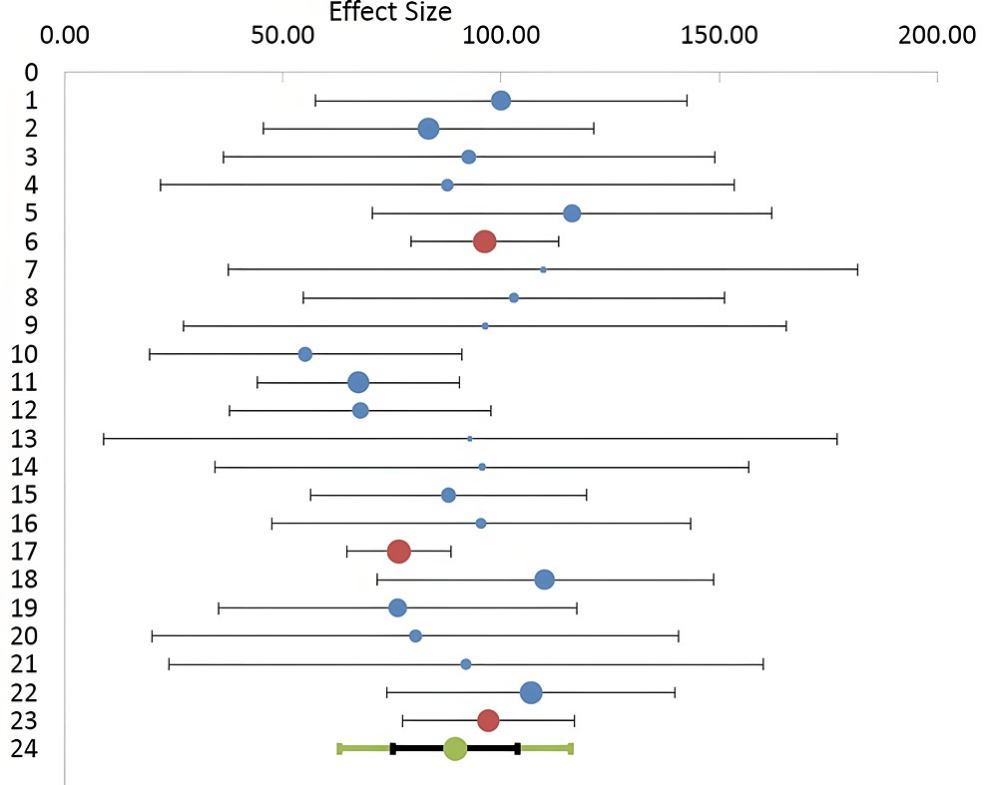
Subgroup analysis and related forest plots to identify the cause of heterogeneity of the reports of %butyrylcholinesterase activity in pregnant women with preeclampsia (mild, severe and unclassified, respectively) compared to healthy pregnant controls in 20 records from 15 studies.

Visual inspection of the constructed funnel plot indicated no publication bias since the effect size points were almost symmetrically distributed in the designated area of the plot, with no imputed points (Figure 4). However, slight asymmetrical points appeared on the left side of the contour-enhanced funnel plot. Based on this notion, Egger’s regression analysis of the records of the studies covered by the meta-analysis showed a significant publication bias effect (t-test= 2.49, p= 0.023). Additional subgroup analysis of the records on PE BChE data that included mild, severe, or unclassified PE confirmed only a low-level heterogeneity among the PE groups (pseudo R^2^= 30.72%). The corresponding Q values (with p values) for the three subgroups of PE were 1.42 (p= 0.840), 2.28 (p= 0.684) and 7.56 (p= 0.579), respectively. Furthermore, the effect size (% BChE) and the % weight of each subgroup (mild, severe, and unclassified PE) as revealed by the forest plot of the subgroups were 96.28% (33.58%), 97.08% (30.73%), and 76.62% (35.69%), respectively (Figure 5). The related values of the I^2^ index, T^2^, and T remained at zero for the three subgroups. It was interesting to note that excluding the mild and severe subgroups of PE, the generally accepted PE in the studies without further classification revealed considerable reduction (23.38%) of BChE activity (effect size 76.62% with 95% C.I. of 64.70, 88.55).

**Figure 6:**
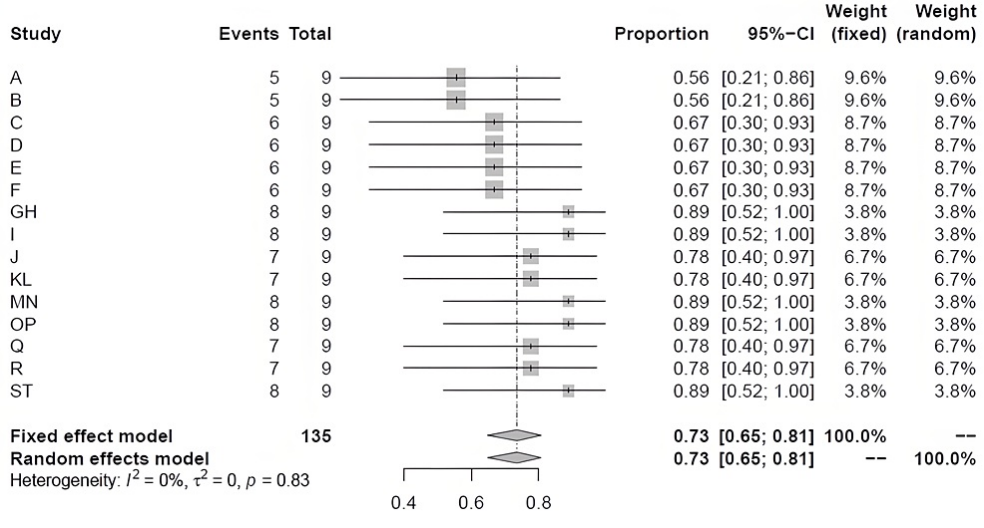
Forest plot of Newcastle–Ottawa Scale scores of the studies

The median score of the 15 studies included in the meta-analysis was 7 ranging between 5 and 8 (Table 3). Examining the total NOS score for each study indicated low quality (score of 5) in two studies, medium quality (score of 6) in four studies, and high quality (score of 7 or 8) in nine studies (Table 3). Furthermore, subjecting the scores of the 15 studies to one-group proportions meta-analysis, produced a forest plot with a standardized proportion mean and 95% C.I. of 0.735 (0.6485, 0.8065) with no heterogeneity (Q= 9; p= 0.8309; I^2^= 0%) (Figure 6).

**Table 3:** Evaluation scores of studies included in the meta-analysis, using the Newcastle–Ottawa Scale (NOS) to detect risk of bias *=Yes

Author [reference No.]	Criteria								Total NOS score
	Selection				Comparability	Exposure			
	1*	2*	3*	4*	**	1*	2*	3*	9
Parviainen et al., 1950 [29]	-	*	-	*	*	*	*	-	5
Pritchard, 1955 [31]	-	*	-	*	*	*	*	-	5
Robertson, 1966 [32]	*	*	-	*	*	*	*	-	6
Satyanarayana, 1986 [33]	-	*	*	*	*	*	*	-	6
Kambam et al., 1987 [34]	-	*	*	*	*	*	*	-	6
Kambam et al., 1988 [17]	-	*	*	*	*	*	*	-	6
Garmendia et al., 1997 [35]	*	*	*	*	**	*	*	-	8
Mahmoud et al., 2003 [36]	*	*	*	*	**	*	*	-	8
Harma et al., 2004 [23]	*	*	*	*	*	*	*	-	7
Osinubi et al., 2009 [24]	*	*	*	*	*	*	*	-	7
Kurdoglu et al., 2012 [37]	*	*	*	*	**	*	*	-	8
Rahimi et al., 2013 [38]	*	*	*	*	**	*	*	-	8
Kharb et al., 2016 [39]	*	*	*	*	*	*	*	-	7
Inangil et al., 2016 [40]	*	*	*	*	*	*	*	-	7
Chen et al., 2022 [11]	*	*	*	*	**	*	*	-	8

Discussion

The main finding of the present meta-analysis is a significant reduction of BChE activity in women with PE (15.16%) compared to healthy pregnancy. This finding was neither associated with significant heterogeneity (Q= 16.26, p= 0.64; I^2^= 0%) nor publication bias (Figure 4). It was unfortunate that not all the studies included in the present meta-analysis examined PE and BChE activity according to the severity of the condition (Table 2). Nonetheless, subgroup examination did not suggest any significant change in the effect size. The effect size values of the mild, severe, and unclassified PE were all below 100% of normal pregnancy (Figure 5). In addition, our results appeared to be robust as the % weights of the records in the meta-analysis ranged from 1.23% to 18.08%. To further add to the credibility, of the 15 studies included in the present meta-analysis, the NOS quality assessment indicated that most of the studies were relatively of high quality (NOS score of 7 or 8 in 9 studies) and the forest plot (Figure 6) showed an effect size of 0.735 (C.I. 0.6485, 0.8065; no heterogeneity).

A reference range of BChE activity in women with PE could not be established from the present results. This is because the studies included in the present analysis have used different methods of ChE determination using either plasma, serum, or whole blood (Table 2). Furthermore, different units have been employed in reporting the enzyme activity, an obstacle we have overcome by converting the BChE activity in PE to % of that of the healthy pregnant group in each study. Future studies should address this point and unify the reporting system of ChE activity during PE.

Any change in BChE activity during pregnancy, especially when complicated with PE, is important in the light of involvement of BChE in the metabolism of neuromuscular agents [13,14]. It was found that prolonged neuromuscular blockage is associated with reduced ChE activity [44,45]. Further, ChE deficiency adversely affects the response of the patient to certain local anesthetics (e.g., procaine) and general anesthetics (e.g., propanidid) [45,46]. It should be stressed here that BChE decreases in activity during normal pregnancy [14,16] and an added burden is exerted if the patient suffers from PE as manifested by further reductions in the enzyme activity [17,24,37-39]. Such a condition during pregnancy with or without PE has also toxicological implications upon exposure to ChE-inhibiting pesticides [47], as well as therapeutic considerations with neuromuscular blocking agents when needed during cesarean section delivery [13,18].

The reason for the reduced BChE activity is not clear at present. As pregnancy and PE were reported to be associated with oxidative stress and/or reduced BChE activity [14-17,38], it was suggested that this enzyme is associated with the pathogenesis of PE through complex pathways involving lipid and lipoprotein metabolism and the occurrence of oxidative stress [38]. This condition has also therapeutic considerations with antioxidants, antiChE agents, and anesthetics which could be used during pregnancy complicated with PE [40,44-46].

In spite of the 15.16% significant reduction in BChE activity in preeclamptic women (Figure 2), it is not clear at present whether the enzyme activity can be used as a biomarker in pregnant women with PE. This is due to the fact that the reduction is only 15.15% as found in the present meta-analysis, and there are discrepancies in the literature regarding the extent of changes in activity expected in pregnancy with PE (reviewed in Table 2). It is possible that the determination of BChE activity during PE could be of value for assessment and follow-up, especially in the initial stages of monitoring and evaluation of the hypertension condition, when used collectively with other biomarkers such as liver enzymes, blood biochemical measurements, and oxidative stress markers [5-11,48].

Limitations of the study

The studies were not randomized because of the nature of the condition (PE) reported. The methods and units of ChE activity measurements were not uniform. No consistent confounders were reported in women with PE in the 15 studies. Unrecognized confounders in the present meta-analysis remain obscure. The stage of pregnancy in relation to PE and the outcome of BChE were not clear from the present study. It was unfortunate to eliminate records from the current meta-analysis that did not include ChE in normal pregnancy so that PE can be compared with [10]. In another study serum ChE activity could not be extracted from the data, but it was reported that reduced enzyme activity was prevalent in PE by 33.3% compared to 6.6% in normal pregnancy [49]. These limitations should form the basis of future studies in biomonitoring BChE activity in pregnancy complicated with PE. The studies should be randomized to examine BChE activity in PE, together with the stage of pregnancy and PE severity in comparison to a matched healthy pregnancy.

## Conclusions

The present meta-analysis is the first attempt of its kind associating BChE activity in pregnant women with PE compared to healthy pregnancy. Measurement of BCHE could be an added biomarker to examine and monitor pregnancy complicated with PE. Although the outcomes suggest that PE could be associated with significantly reduced BChE in comparison with normal pregnancy, the present status of reduced BChE activity (15.16%) in the plasma or serum could not be implicated as a biomarker of PE in women. However, examining BChE activity could be collectively used with other biomarkers to monitor PE cases.
